# Peritoneal tuberculosis

**DOI:** 10.1007/s15010-025-02677-8

**Published:** 2025-10-31

**Authors:** Laura Tervo, Artturi Mäkinen, Heikki Tuominen, Juha Rannikko

**Affiliations:** 1 Department of Internal Medicine, Tampere University Hospital, Box 2000, Tampere, FI- 33521 Finland; 2 Faculty of Medicine and Health Technology, University of Tampere, Box 100, Tampere, FI- 33014 Finland; 3 Fimlab Laboratories, Department of Pathology, Tampere University Hospital, Box 66, Tampere, FI-33520 Finland; 4 Department of Clinical Physiology and Nuclear Medicine, Tampere University Hospital, Box 2000, Tampere, FI-33521 Finland

**Keywords:** Tuberculosis, PET imaging, PCR, Granulomatous inflammation

A 74-year-old Finnish-born woman with a history of nummular eczema presented to the emergency department with fever and abdominal pain. Computed tomography revealed ascites and cholecystitis. The inflamed gallbladder was removed laparoscopically. During the operation, the peritoneal surface was observed to be covered with nodules, and a biopsy was taken. Histopathological analysis showed necrotizing granulomas; however, polymerase chain reaction (PCR), Ziehl-Neelsen staining, and cultures were negative for tuberculosis. Subsequently, new biopsies and fluid samples were collected from ascites, pleural fluid, an axillary lymph node, and via endobronchial ultrasound; all were culture- and PCR-negative for tuberculosis and histologically negative for malignancy. Only the histological sample taken from the axilla showed granulomas and a structure that could represent a single tuberculosis bacillus (Fig. 1, 2). Positron emission tomography (PET) revealed extensive peritoneal inflammation and some active lymph nodes (Fig. 3C1-3C4). Empiric four-drug tuberculosis therapy was initiated. Finally, both PCR and culture from a cervical lymph node confirmed tuberculosis, which was sensitive to first-line drugs. After six months of treatment, the inflammation had almost resolved (Fig. 3C5-3C8) except for a small focus of persistent inflammatory metabolic activity on the caudal surface of the liver and in the liver parenchyma. Some studies suggest that persistent inflammation on follow-up PET scans is associated with a higher risk of tuberculosis relapse [1, 2]. Due to this and especially the widespread nature of the disease, treatment was extended to 9 months. This case also illustrates the challenges of diagnosing tuberculosis, as five separate samples were required before the diagnosis was confirmed.

**Fig. 1:**
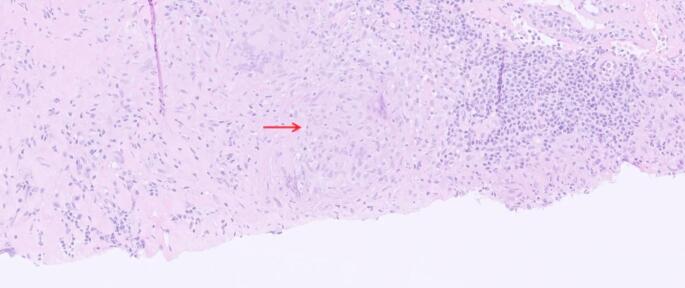
Histologically, the lymph node biopsy revealed sarcoid-like granulomas (red arrow) composed of epithelioid histiocytes and multinucleated Langhans-type giant cells

**Fig. 2:**
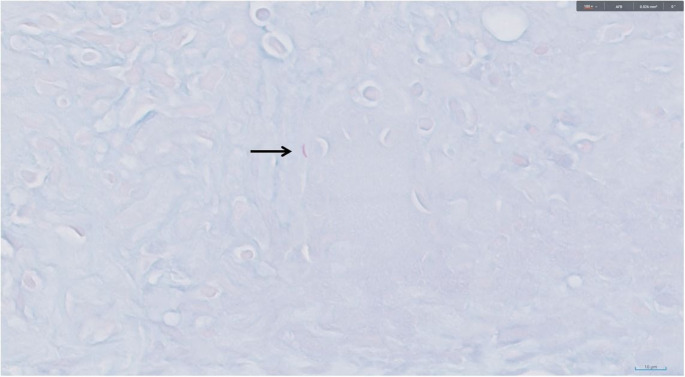
A single acid-fast, mycobacteria-like organism (black arrow) was identified in the granulomas using Ziehl –Neelsen staining

**Fig. 3:**
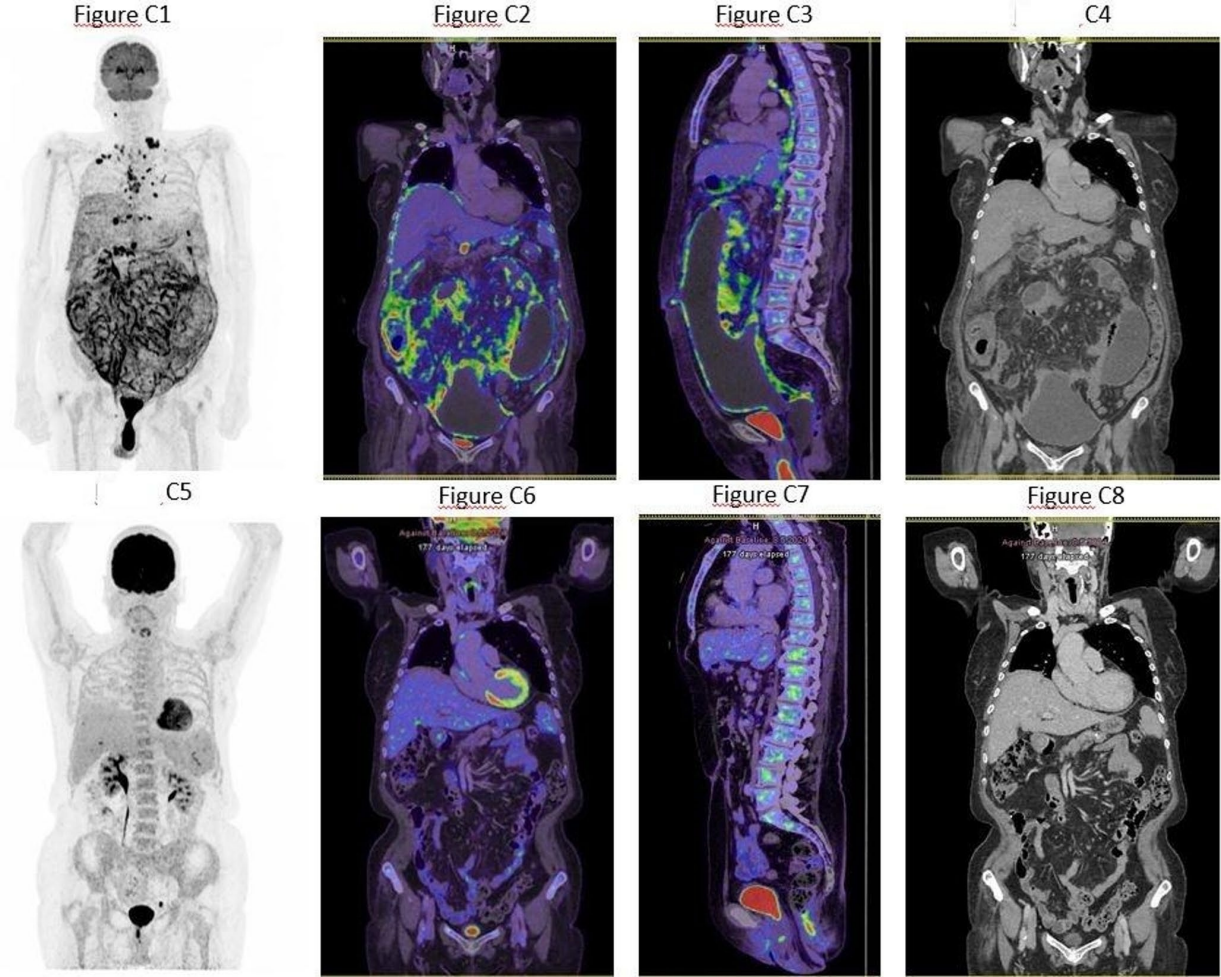
FDG-PET / contrast-enhanced CT Images at baseline (upper row) and after treatment (lower row). Maximum Intensity Projection (MIP) at baseline showing diffuse metabolic activity in lymph nodes of the neck, mediastinum, and epigastrium. Diffuse hypermetabolism is seen in the intra-abdominal cavity along the mesentery and peritoneum (**C1**). After treatment, complete resolution of supradiaphragmatic lymph nodes is observed. A new active focus in the liver parenchyma and low-grade persistent metabolic activity on the caudal surface of the liver are noted, while other peritoneal and mesenteric foci have disappeared (**C5**). Diffuse mesenteric and peritoneal wall hypermetabolic changes and a good treatment response are visualized in coronal and sagittal fusion images (**C2**, **C3**, **C6**, **C7**). Contrast-enhanced CT shows nodulation, diffuse mesenteric edema, and ascites, as well as their resolution after treatment (**C4**, **C8**)

## Data Availability

No datasets were generated or analysed during the current study.
